# The magnitude and associated factors of childbirth fear among pregnant women attending antenatal care at public hospitals in Ethiopia: a cross-sectional study

**DOI:** 10.1186/s12884-022-04544-y

**Published:** 2022-03-19

**Authors:** Robera Demissie Berhanu, Asresash Demissie Abathun, Endalew Hailu Negessa, Lensa Gari Amosa

**Affiliations:** 1 Department of Nursing, College of Health Sciences, Mettu University, P.O.Box: 318, Mettu, Ethiopia; 2 School of Nursing, Institute of Health Sciences, Jimma University, Jimma, Ethiopia; 3 Department of Midwifery, College of Health Sciences, Mettu University, Mettu, Ethiopia

**Keywords:** Pregnant women, Childbirth fear, Ethiopia

## Abstract

**Background:**

Childbirth fear affects 5–40% of all mothers around the world, and there is mounting evidence that it has harmful impacts on women’s health. It could potentially lead pregnant women to feel isolated and unsupported if not identified. But studies addressing this issue are limited in Ethiopia. Therefore, this study was aimed at assessing the magnitude and associated factors of childbirth fear among pregnant women attending antenatal care at public hospitals in West Wollega Zone.

**Methods:**

Facility-based cross-sectional study was conducted among 304 pregnant women selected by systematic random sampling from 20 March to 20 April 2020. A structured interviewer-administered questionnaire was adapted and used to collect data. Data were entered into EpiData version 3.1 and exported to IBM SPSS statistics version 26 for analysis. Descriptive statistics were done to calculate frequencies, mean scores, and standard deviation. Bivariate and multivariable logistic regression was used to identify factors associated with childbirth fear. Variables with *p* < 0.25 in bivariate analyses were selected for multivariable analysis. Finally, statistical significance was declared at *p* < 0.05.

**Results:**

Out of the total of 304 participants, 298 completed the interview making the response rate 98%. The overall prevalence of childbirth fear was 28.9% with 95% CI (23.5, 34.2). Mean age of the respondents was 27.60 (SD ± 4.56) years. Having previous pregnancy complications [AOR (95% CI)], [6.949 (2.060 – 23.445), presence of long time during childbirth [AOR (95% CI)], [4.765 (1.161 – 19.564)], presence of episiotomy [AOR (95% CI)], [4.197 (1.107 – 15.917)], low social support [AOR (95% CI)], [.011 (.003 – .050)] were significantly associated with childbirth fear.

**Conclusion:**

Pregnant women in the study area have a significant level of childbirth fear. Previous pregnancy complications, prolonged labor, labor pain, previous perineal tear, and social support were all found to be significantly linked with childbirth fear. This calls for the need to identify and develop interventions for women to reduce childbirth fear during pregnancy.

## Introduction

Childbirth is multidimensional physiologic process that is unique to each woman, still strongly influenced by her social context [1]. Childbirth fear (CBF) is a negative perception that starts during pregnancy and persists through birth and the postpartum period. It is a pivotal event in every woman's life, in both in nulliparous and multiparous women, affecting women and their families on a mental, social, and physiological level [2, 3]. In nulliparous women, fear arises from unknown, pain, and loss of control. In parous women, fear arises from previous experiences such as poor perinatal outcomes, traumatic births, and prospect of the future [4–6]. Childbirth fear can sometimes be so high that it inhibits women from becoming pregnant and even interferes with their daily lives [2].

CBF ranges from almost total absence of fear to extreme fear. As the level fear increases its consequence becomes severe [7–9]. Pregnant women affected with childbirth fear are at increased risk for difficult, prolonged and upsetting labour. In addition, CBF has been associated with adverse postpartum mental health difficulties [3, 10]. Many studies reported that CBF could be a reason for deciding to have cesarean section for delivery when there is no medical cause for it. Higher childbirth fear during third trimester of pregnancy increases the likelihood of choosing cesarean-Section [11–18]. Over recent decades, worldwide caesarean rate has continuously increased despite the fact that it is associated with higher complications [19].

Even though maternity care in high-income countries is safe, CBF is a prevalent problem. It affects 5–40% of all mothers across the world [20–25]. A study shows that the prevalence of CBF varies from 4.5% to 15.6% in six European countries [26]. According to a study conducted in Arba Minch town, southern Ethiopia, 25.3% and 24.5% of women are affected by high degree and severe degree fear, respectively [27]. Another study in Jinka Town, Southern Ethiopia shows childbirth fear affects 24.2% of pregnant mothers [28]. Several studies have found that past pregnancy complications, past experience of delivery complications, and presence of strong social support increase the likelihood of experiencing childbirth fear [22, 26, 27, 29, 30]. Therefore, prompt and timely management of this complications and promoting maternal health are crucial [8, 22]. As a result, providing pregnant mothers with a secure atmosphere in which to receive both mental and physical care from their family as well as from health institutions is one strategy to address this problem [22, 26]. In Ethiopia, the information required to provide this is very limited, and this issue has not been assessed yet in the area where the present study was done. Understanding the magnitude and factors influencing childbirth fear is thus important in improving women's health. The findings from the study may have implications for midwives/health care providers and policymakers in terms of improving women’s awareness and providing evidence-based practice. Therefore, this study was aimed to assess the magnitude and associated factors of childbirth fear among pregnant women attending antenatal care at public hospitals in West Wollega Zone.

## Materials and methods

### Study design, setting, and period

A facility-based cross-sectional study was conducted at public hospitals in West Wollega Zone from 20 April to 20 May 2020. West Wollega Zone is one of the zones in the Oromiya region of Ethiopia. Based on the 2007 Census conducted by the Central Statistical Agency of Ethiopia, this zone has a total population of 1,350,415, of whom 671,538 are men and 678,877 women; with an area of 10,833.19 square kilometers. The zone has a population density of 124.66. There are five public hospitals in the zone namely Gimbie General Hospital, Nedjo General hospital, Begi General hospital, Mendi General Hospital, and Bube primary hospital. In these hospitals, the monthly estimated number of pregnant visiting ANC care is 1792 and the estimated number of deliveries attended by the skilled health care provider is 8372.

### Study population and recruitment criteria

The study population for this study was all pregnant women were attending antenatal care at public hospitals in West Welega Zone. All pregnant women were attending antenatal care at public hospitals in West Welega Zone were included in the study. Pregnant women who are disabled (hearing and speaking difficulty) and/or critically ill were excluded from the study.

### Sample size determination

Sample size was calculated using a formula for a single population proportion considering confidence interval of 95% (Z = 1.96), margin of error 5% (*d* = 0.05), and population proportion of 50% (*p* = 0.5).

$$n= \frac{{Z (\alpha\left/ 2\right.)}^{2}\times p(1-p)}{{d}^{2}}$$

$$n= \frac{{(1.96)}^{2}\times 0.5(1-0.5)}{{(0.05)}^{2}}=384$$

Since the estimated total number of pregnant women attending antenatal care (ANC) in the hospitals is 1152, which is less than 10,000; and also since the ratio of initial sample size calculated to the total number of pregnant women attending ANC in the hospitals is greater than 0.05, correction formula was used and sample size became 289.

$$\begin{array}{cc}{n}_{f}=\frac{{n}_{i}}{1+ \frac{{n}_{i}}{N}}& {n}_{f}=\frac{384}{1+ \frac{384}{1152}} = 289\end{array}$$

Finally, 5% nonresponse rate was added and the final sample size became 304.

### Sampling procedure

**Fig. 1:**
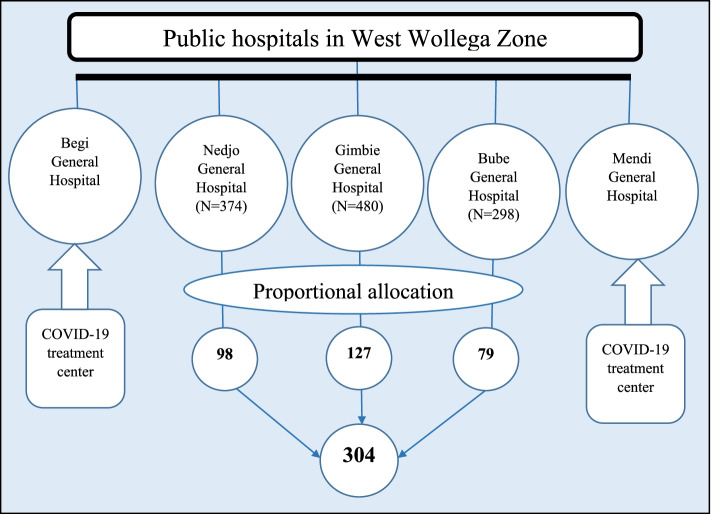
Flowchart for sampling procedure

By the time of data collection, two hospitals were serving as the COVID-19 treatment center. Therefore, the study was conducted at the remaining three hospitals. The calculated sample size was allocated proportionally to each hospital based on the number of each hospital’s monthly caseload derived from their annual ANC caseload. Sampling interval was calculated for each hospital, which was 4. Then the study participants were selected from each hospital by using systematic random sampling technique. Random start was selected by using lottery method on the first day of data collection at each hospital and every 4^th^ pregnant woman visiting each hospital for ANC was interviewed (Fig.1).

### Variables and measurement

#### Sociodemographic variables

Age, marital status, religion, educational status, occupation, and family size.

#### Obstetric variables

Age at marriage, current gestational age, parity, gravida, number of ANC visit, gestational age at first ANC visit, previous pregnancy complication, and mode of delivery.

#### Previous experience

Prolonged labour, labour pains, Loss of bowel and/or bladder control, excessive bleeding during childbirth, fear of death, previous perineal tear, and previous caesarian section.

#### Childbirth fear

Is measured using 12 items on a 7-point Likert scale with the value ranging from 12– 84. Participants who scored mean score and above were considered as having high childbirth fear and those who scored below the mean score were considered as having low childbirth fear.

#### Social support

Measured using 12 items on a 7-point Likert scale with the value ranging from 12– 84. Study participants who scored mean score and above were considered as having high social support and those who scored below the mean score were considered as having low social support.

#### Critically ill pregnant women

Pregnant women who had conditions which prevent them from properly responding to the interviews such as severe pre-eclampsia, eclampsia, and antepartum haemorrhage.

### Data collection

Data were collected by six BSc midwives recruited from each hospital. Three BSc midwives were assigned from each hospital and supervised data collection processes. Data collector made exit interview with the study participants keeping all the precautions for the COVID-19 prevention. Data collection processes were continuously monitored by the principal investigators.

### Study instruments

A structured interviewer-administered questionnaire was adapted from related studies [31–34] and used. The questionnaire was first prepared in English then translated to Afaan Oromo and back-translated to English by language experts to maintain its consistency. The questionnaires had five parts: socio-demographic characteristics, obstetrical details, Childbirth Fear Scale, previous experience related questionnaire, and Multidimensional Scale of Perceived Social Support. The scales used to assess childbirth fear and perceived social support contain 7-point Likert-type questions with responses ranging from very strongly disagree (1) to very strongly agree (7). In the current study, the Cronbach’s alpha were 0.86, 0.78, and 0.81 for childbirth fear questionnaire, previous experience related questionnaire, social support questionnaire, respectively.

### Data quality management

Pretest was conducted on 5% (15) of the sample size at Mendi primary hospital. Data collected for the pretest was analyzed and used for amending data collection tool. Then reliability of the data collection tool was measured, data collection time was estimated and modifications such as logical order and rewriting items difficult to understand were made as well. One-day training was given for data collectors and supervisors regarding the objective of the study, data collection tool, and procedures and how to approach respondents. The quality, consistency and completeness of data were kept through careful collection and supervision on daily basis.

### Data analysis

Data were coded and entered into EpiData Version 3.1 and exported to IBM SPSS Statistics Version 26.0 for analysis. Then data were analyzed using descriptive statistics such as frequencies, mean scores, and standard deviations. Bivariate binary logistic regression was used for selecting candidate variables for multivariable binary logistic regression. Accordingly, all independent variables with $p<$0.25 were considered candidates for multivariable binary logistic regression. Finally, statistical significance was declared at $p<$0.05 in the multivariable binary logistic regression. The fitness of the model was checked by Hosmer and Lemeshow’s test, which was found to be insignificant (*p* = 0.253) indicating that the model was fitted. Backward logistic regression was used for selecting variables in the final model and *p*-value for the model was $<$ 0.001. The strength of statistical association was measured by adjusted odds ratios and 95% confidence intervals.

## Results

Out of the total sample, 298 of the study participants were involved in this study giving a response rate of 98.03%.

### Socio-demographic characteristics

The mean age of the respondents was 27.60 (SD ± 4.56) years, majority of them 162 (54.4%) fell into the age group of 25–29 years. The vast majority of the respondents 273(91.6%) were married, and 105(35.2%) were housewives. About 93 (31.2%) of respondents attended primary education (Table 1).

**Table 1:** Socio-demographic characteristics of pregnant women attending ANC at public hospitals in West Wollega Zone, 2020 (*n* = 298)

Variables	Category	Frequency	Percentage
**Age** M = 27.60 SD = $\pm$ 4.56	15 – 19 years	13	4.4
	20 – 24 years	41	13.8
	25 – 29 years	162	54.4
	30 – 34 years	58	19.5
	35 + years	24	8.1
**Marital status**	Married	273	91.6
	Divorced	14	4.7
	Widowed	11	3.7
**Religion**	Muslim	59	19.8
	Protestant	184	61.7
	Orthodox	47	15.8
	Catholic	8	2.7
**Educational status**	Cannot read and write	59	19.8
	Can read and write	20	6.7
	Primary school	93	31.2
	Secondary school	71	23.8
	College	34	11.4
	University	21	7.0
**Occupation**	Self-employed	45	15.1
	Government employed	56	18.8
	Daily laborer	73	24.5
	Housewife	105	35.2
	NGO	19	6.4
**Family size**	≤ 3	100	33.6
	4 – 5	163	54.7
	≥ 6	35	11.7

*NGO* Non-governmental organization, *SD* Standard deviation

### Obstetric details of participants

Majority of the respondents (83.9%) were married when they were in the age group of 18 – 24 years. About two-third of respondents’ gestational age was greater than or equal to 28 weeks at the time of data collection. For the vast majority of the women (96.6%), the total number of gravida was in between 2 and 4 and majority of them (85.5%) had number of para less than or equal to 2 (Table 2).

**Table 2:** Obstetric details of pregnant women visiting attending ANC at public hospitals in West Wollega Zone, 2020 (*n* = 298)

Variables	Category	Frequency	Percentage
Age at marriage	$<$ 18 years	39	13.1%
	18 – 24 years	250	83.9%
	$\ge$ 25 years	9	3%
Current gestational age	$\le$ 28 weeks	204	68.5
	$>$ 28 weeks	94	31.5
Gravida	2 – 4	288	96.6
	$\ge$ 5	10	3.4
Para	$\le$ 2	255	85.6
	3 – 4	43	14.4
Previous pregnancy complication	Yes	102	34.2
	No	196	65.8
Number of ANC received during current pregnancy	$<$ 4	247	82.9
	$\ge$ 4	51	17.1
Gestational age when you first received antenatal care for this pregnancy	$<$ 16	207	69.5
	$\ge$ 16	91	30.5
Mode of delivery	SVD	135	45.3
	Cesarean section	74	24.8
	IAVD	89	29.9

*ANC* Antenatal care, *SVD* spontaneous vaginal delivery, *IAVD* Instrumental assisted vaginal delivery

### Previous experience and social support

Among the study respondents, 19.5% of them reported they experienced prolonged labour while 18.5% of them experienced labour pain. Eighty-two (27.5%) of them had experience of loss of bowel and/or bladder control while 42 (14.1%) of them had previous excessive bleeding during childbirth. About two-third (67.4%) of them had low social support (Table 3).

**Table 3:** Previous experience and social support of pregnant women attending ANC at public hospitals in West Wollega Zone, 2020 (*n* = 298)

Variables	Category	Frequency	Percentage
Prolonged labour	Yes	58	19.5
	No	240	80.5
Labour pains	Yes	55	18.5
	No	243	81.5
Loss of bowel and/or bladder control	Yes	82	27.5
	No	216	72.5
Excessive bleeding during childbirth	Yes	42	14.1
	No	256	85.9
Previous perineal tear	Yes	77	25.8
	No	221	74.2
Previous caesarian section	Yes	63	21.1
	No	235	78.9
Social support	High	94	31.5
	Low	201	67.4

### Magnitude of childbirth fear

**Fig. 2:**
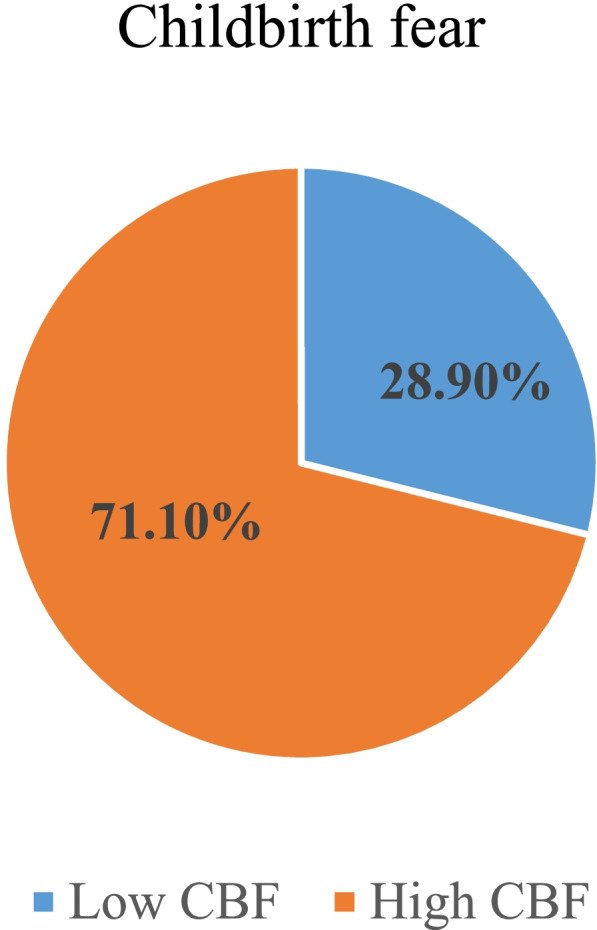
Pie chart showing magnitude of childbirth fear among pregnant women attending ANC at public hospitals in West Wollega Zone, 2020 (*N* = 298)

From the 298 respondents interviewed, 86(28.9%) (95% CI (23.5, 34.2)) experienced high childbirth fear among pregnant women attending antenatal care at public hospitals (Fig. 2).

### Trimester-based distribution of childbirth fear

High childbirth fear was reported by 64% of pregnant women in their second trimester, but only 36% of those in their third trimester (Table 4).

**Table 4:** Trimester-based distribution of childbirth fear of pregnant women attending ANC at public hospitals in West Wollega Zone, 2020 (*n* = 298)

	**Child birth fear**			**Total**
		Low	High	
**Trimesters of pregnancy**	Second trimester of pregnancy	101 (67.6%)	55(64%)	156
	Third trimester of pregnancy	111(52.4%)	31(36%)	142
**Total**		212	86	298

Because none of the participants began antenatal follow-up during the first trimester of pregnancy, the first trimester is not included in the distribution

### Factors associated with childbirth fear

Both bivariate and multivariable binary logistic regression analyses were carried out to identify factors associated with childbirth fear. Bivariate analyses showed marital status, occupation and family number, obstetric related factors like age at marriage, current gestational age, previous pregnancy complications, number of ANC received during current pregnancy, gestational age when first received ANC, and mode of delivery were significantly associated with childbirth fear at *p* < 0.25. Bivariate analyses also showed that five previous experience-related factors (prolonged, labour pains, excessive bleeding during childbirth, previous perineal tear, and previous caesarian section) and social support were significantly associated with childbirth fear at  *p* < 0.25. All significant in the bivariate analyses were entered into the multivariable logistic regression analysis to identify factors associated with childbirth fear.

Based on the finding from multivariable binary logistic regression, pregnant women who had previous pregnancy complications were 7 times more likely to experience childbirth fear as compared to those who did not have [AOR (95% CI)], [6.949 (2.060 – 23.445)], (*p* = 0.002). On the other hand, pregnant women who had prolonged labour previously were 5 times more likely to experience childbirth fear as compared to those who had not [AOR (95% CI)], [4.765 (1.161 – 19.564)], (*p* = 0.030). Similarly, pregnant women who had labour pain previously were 5 times more likely to experience childbirth fear as compared to those who had not [AOR (95% CI)], [4.840 (1.067 – 21.957)], (*p* = 0.041). Additionally, pregnant women who experienced perineal tear were 4 times more likely to experience childbirth fear as compared to those who did not [AOR (95% CI)], [4.197 (1.107 – 15.917)], ( *p* = 0.035). Pregnant women who had high social support were 98.9% times less likely to experience childbirth fear than those who had not [AOR (95% CI)], [0.011 (0.003 – 0.050)], ( *p* = 0.000). Accordingly, previous obstetric complication, prolonged labour, labour pain, previous perineal tear, and social support were associated with childbirth fear (Table 5).

**Table 5:** Multivariable logistic regression analysis for factors associated with childbirth fear among pregnant women attending ANC at public hospitals in West Wollega Zone, 2020 (*n* = 298)

Variables	Childbirth fear		COR (95% C.I.)	AOR (95% C.I.)	*P* value
	**Low**	**High**			
**Marital status**					
Married®	196(71.8%)	77(28.2%)			
Widowed	10(90.9%)	1(9.1%)	3.394 (1.140, 10.103)	1.840 (.174, 19.397)	612
Divorced	6(42.9%)	8(51.7%)	0.255 (0.032, 2.022)	2.601 (.207, 32.707)	.459
**Occupation**					
Self-employed®	32(71.1%)	13(28.9%)			
Gov’t employed	50(59.3%)	6(10.7%)	7.312 (.883, 60.577)	.209 (.023, 1.876)	.162
Daily laborer	41(56.2%)	32(43.8%)	2.160 (0.243, 19.194)	1.507 (.223, 10.183)	.674
Housewife	71(67.6%)	34(32.4%)	14.049 (1.780, 110.89)	.355 (.058, 2.180)	.263
NGO	18(94.7%)	1(5.3%)	8.620 (1.104, 67.275)	.251 (.010, 6.445)	.404
**Family number**					
≤3	69(69.0%)	31(31.0%)	2.171 (.818, 5.762)	1.456 (.213, 9.945	.702
4 – 5	114(69.9%)	49(30.1%)	2.077 (0.811, 5.322)	1.162 (.205, 6.594)	.866
≥ 6®	29(82.9%)	6(17.1%)			
**Age at marriage**					
<18	32(82.1%)	7(17.9%)	1.750 (0.187, 16.339)	.061 (.003, 1.385)	.079
18 – 24	172(68.6%)	78(31.2%)	3.628 (0.446, 29.508)	.141 (.010, 1.934)	.143
≥ 25®	8(88.9%)	1(11.1%)			
**Previous pregnancy complications**					
Yes	38(37.3%)	64(62.7%)	.075 (0.041, 0.137)	6.949 (2.060,23.445)	.002*
No®	174(88.8%)	22(11.2%)			
**GA at first ANC?**					
<16®	141(68.1%)	66(31.9%)			
≥16	71(78.0%)	20(22.0%)	0.393 (0.213, 0.723)	1.204 (.289, 5.009)	.799
**Mode of delivery**					
SVD®	92(68.1%)	43(31.9%)			
Caesarian section	58(78.4%)	16(21.6%)	1.075 (0.601, 1.915)	2.955 (.592, 14.747)	.187
IAVD	62(69.7%)	27(30.3)	.633 (0.310, 1..294)	.809 (.226, 2.893)	.745
**Prolonged labour**					
Yes	25(43.1%)	33(56.9%)	4.657 (2.549, 8.508)	4.765 (1.161,19.564)	.030*
No®	187(77.9%)	53(22.1%)			
** Labour pain**					
Yes	13(23.6%)	42(76.4%)	14.612 (7.238, 29.500)	4.840 (1.067,21.957)	.041*
No®	199(81.9%)	44(18.1%)			
**Excessive bleeding during childbirth**					
Yes	26(61.9%)	16(38.1%)	1.635 (0.828, 3.230)	.887 (.198, 3.967)	.876
No®	186(72.7%)	70(27.3%)			
**Previous perineal tear**					
Yes	27(31.2%)	53(68.8%)	12.581 (6.851, 23.102)	4.197 (1.107,15.917)	.035*
No®	188(85.1%)	33(14.9%)			
**Previous caesarian section**					
Yes	36(57.1%)	27(42.9%)	2.237 (1.253, 3.994)	0.404 (.092, 1.774)	0.230
No®	176(74.9%)	59(25.1%)			
**Social support**					
High	17(17.5%)	80(82.5%)	0.007 (0.002, 0.017)	.011 (.003, .050)	.000*
Low®	195(97.0%)	6(3.0%)			

Dependent variable: Childbirth fear *Significant at *p* < 0.05. ®Reference

*ANC* Antenatal care, *GA* Gestational age, *NGO* Nongovernmental organization, *SVD* spontaneous vaginal delivery, *IAVD* Instrumental-assisted vaginal delivery

## Discussion

This study was aimed to assess childbirth fear and associated factors at public hospitals of West Wollega Zone. The study finding indicates that the magnitude of CBF was 28.9% with 95% CI (23.5, 34.2). This finding is in line with the findings from the studies conducted in Southern Ethiopia and Australia where the prevalences of childbirth fear were reported to be 24.2 and 31.5% respectively [28, 35]. However, it is lower than the finding from a study conducted in Ireland, in which the prevalence of childbirth fear was 36.7% [35, 36]. On the other hand, the finding is higher than that of a study conducted in Malawi, in which the prevalence of CBF was found to be 20% [30]. The disparity in prevalence of childbirth fear could be attributed to differences in antenatal and delivery care quality. Furthermore, differences in the prevalence of childbirth fear may be due to sociocultural differences. People from different cultures may have varied views and attitudes toward childbirth [37, 38], which may contribute to differences in the prevalence of childbirth fear.

Variables associated with childbirth fear were also investigated in this study. Previous pregnancy complications, prolonged labour, labour pain, perineal tear, and social support were significantly associated with childbirth fear.

The finding from this study indicates that pregnant women who had previous pregnancy complications were 7 times more afraid of giving birth as compared to those who did not experience complications. This is supported by a similar finding from a study in Sweden which found significant association between previous birth complication and childbirth fear [22]. The current study’s findings also indicates prolonged labor was significantly associated with childbirth fear. The odds of childbirth fear was 5 times higher among pregnant women who had previous experience of prolonged labour compared to those who did not experience. This finding is consistent with a study conducted in six European countries including Belgium, Iceland, Denmark, Estonia, Norway, and Sweden, which found significant association between prolonged and childbirth fear [26]. The finding of the present study also indicates that previous perineal tear was significantly associated with childbirth fear. The odds of childbirth fear was 4 times higher among pregnant women who had previous perineal tear than those who did not have. Similarly, a study in Slovenia reported that episiotomy was associated with CBF [29]. It is also consistent with the World health organization report which shows perineal tearing may lead to childbirth fear [21]. The possible reasons for such similarity may be because mothers remember past traumas or feelings and they might think that they face pregnancy complications, prolonged labour, or perineal tear again.

The study result also indicates that having labour pain was significantly associated with childbirth fear. The findings of the studies in Malawi, Ireland, and Sweden support this conclusion [25, 30, 36]. The odds of childbirth fear was 5 times higher among pregnant women who had labour pain previously compared to those who had not. Childbirth is a natural event accompanied by labor pains and possibly unpredictable components of the process may partly explain why women in different parts of the world share similar factors linked with childbirth fear [39, 40]. Overall, health care providers should pay careful attention in providing mothers with a suitable atmosphere as most of the factors associated with CBF occur during intrapartum period.

The finding of the present study also indicates that women who received low social support during pregnancy reported higher levels of childbirth fear. The odds of childbirth fear was decreased by 98.9% times among pregnant women who had high social support compared to their counterparts. This finding is similar with previous studies conducted in Arba Minch town, southern Ethiopia, Finland, and Malawi [27, 30, 41]. The possible reason may be pregnant mothers’ attention may be diverted when they get strong support from their family, neighbor, and health care providers. In addition, they may view birthing processes as controllable when they share ideas from families, neighbors, and health care providers.

Since the study was conducted at public hospitals, its findings should not be generalized for pregnant women visiting public health center and for the entire population. The study design was cross-sectional so that cause and effect relationship of variables could be difficult to ascertain.

## Conclusion

Pregnant women in the study area have a significant level of childbirth fear. Previous pregnancy complications, prolonged labor, labor pain, previous perineal tear, and social support were all found to be significantly linked with childbirth fear in the study. The findings of the study inspire regional health bureaus and their partners to work together to enhance treatments aimed at reducing pregnancy complications. It also urges zonal health departments and woreda health offices to ensure that all pregnant women have access to ANC follow-up and education. As a result, complications can be detected and treated early. The findings of the study encourage health care workers to treat labor pains and perineal tear as soon as possible. The study also encourages family members and neighbors to support pregnant women. Large-area which includes public health center, community based study and/or longitudinal study is encouraged.

## Data Availability

Data used and analyzed during the current study are available from corresponding author on reasonable request.

## Abbreviations

ANC: Antenatal care
AOR: Adjusted odds ratio
CBF: Childbirth fear
CI: Confidence interval
COR: Crude odds ratio
COVID-19: Coronavirus disease 2019
C/S: Cesarean section
IAVD: Instrumental assisted vaginal delivery
NGO: Nongovernmental organization
SD: Standard deviation
SPSS: Statistical package for social sciences
SVD: Spontaneous vaginal delivery
